# Supplementary material for: Massively parallel jumping assay decodes Alu retrotransposition activity
Source: Nat Commun. 2025 May 9;16:4310. doi: 10.1038/s41467-025-59347-4 (PMC12062377; doi:10.1038/s41467-025-59347-4)
Supplement: Supplementary file 3 — Description of Additional Supplementary Files [file 41467_2025_59347_MOESM3_ESM.pdf]

Description of Additional Supplementary Files

File Name: Supplementary Data 1
Description: Mutagenesis primers

File Name: Supplementary Data 2
Description: Summary table for mutation spectra in mutagenzed libraries

File Name: Supplementary Data 3
Description: Alu recovery and resurrection primers

File Name: Supplementary Data 4
Description: Alu sx haplotypes. Significant effects were defined using the DESeq2 package with a Wald-test (two-sided).

File Name: Supplementary Data 5
Description: Alu6b haplotypes, Significant effects were defined using the DESeq2 package with a Wald-test (two-sided).

File Name: Supplementary Data 6
Description: Alu14b haplotypes, Significant effects were defined using the DESeq2 package with a Wald-test (two-sided).

File Name: Supplementary Data 7
Description: Aluh1.1 haplotypes, Significant effects were defined using the DESeq2 package with a Wald-test (two-sided).

File Name: Supplementary Data 8
Description: Unique AluS elements with 280 bp intact core in the human genome

File Name: Supplementary Data 9
Description: Alignment of Alus tested with AluSx consensus and human Reference

File Name: Supplementary Data 10
Description: Adaptors Indeces and barcodes

File Name: Supplementary Data 11
Description: Nucleotide position probability analysis in SRP domain in left and right arm of Alu high jumping and lower jumping haplotypes. Absolute probability was calculated based on the observed occurrence of a nucleotide at each position in the Alu haplotypes classified as low or high jumpers. After the DESeq2 analysis, the haplotypes found in each replicates were merged together and considered as one pool to calculate the absolute probabilty is calculated as number of observation of the certain nucleotide over total number of individuals nucleotides at certain position and expressed a numerical from 0 to 1.

File Name: Supplementary Data 12
Description: Number of Haplotypes in each Alu-Mut library. Significant effects were defined using the DESeq2 package with a Wald-test (two-sided) and p-value threshold of 10-5. Classes were defined as

significant high jumpers (Log2FC > 2), significant low jumpers (Log2FC < -2, jumping counts > 0) and non-jumpers (Log2FC < -2, jumping counts = 0 and plasmid count > 50).
